# Nutraceuticals: Focus on Anti-Inflammatory, Anti-Cancer, Antioxidant Properties in Gastrointestinal Tract

**DOI:** 10.3390/antiox11071274

**Published:** 2022-06-28

**Authors:** Giusy Rita Caponio, Tamara Lippolis, Valeria Tutino, Isabella Gigante, Valentina De Nunzio, Rosa Anna Milella, Marica Gasparro, Maria Notarnicola

**Affiliations:** 1 National Institute of Gastroenterology “Saverio de Bellis”, Research Hospital, Castellana Grotte, 70013 Bari, Italy; giusy.caponio@irccsdebellis.it (G.R.C.); tamara.lippolis@irccsdebellis.it (T.L.); valeria.tutino@irccsdebellis.it (V.T.); isabella.gigante@irccsdebellis.it (I.G.); valentina.denunzio@irccsdebellis.it (V.D.N.); 2 Research Centre for Viticulture and Enology, Council for Agricultural Research and Economics, Turi, 70010 Bari, Italy; rosaanna.milella@crea.gov.it (R.A.M.); marica.gasparro@crea.gov.it (M.G.)

**Keywords:** bioactive molecule, gut microbiota, bioavailability, phenolic compounds, polyunsaturated fatty acids

## Abstract

In recent years, nutraceuticals have gained great popularity, owing to their physiological and potential health effects, such as anti-inflammatory, anti-cancer, antioxidant, and prebiotic effects, and their regulation of lipid metabolism. Since the Mediterranean diet is a nutritionally recommended dietary pattern including high-level consumption of nutraceuticals, this review aimed to summarize the main results obtained by our in vitro and in vivo studies on the effects of the major constituents of the Mediterranean diet (i.e., extra virgin olive oil compounds, polyunsaturated fatty acids, and fruit components). Based on experimental studies, the therapeutic purpose of nutraceuticals depends on their bioavailability, solubility, toxicity, and delivery system. This review provides more in-depth knowledge on the effects linked to nutraceuticals administration on human health, focusing the gastrointestinal tract and suggesting specific dietary components for personalized adjuvant therapies.

## 1. Nutraceuticals

The fascinating topic of nutraceuticals has always existed throughout history and further confirmed by modern medicine [1,2,3,4]. The term “nutraceutical”, coined in 1989 by Stephen DeFelice, defines the role of food in providing medical and health benefits [5]. Nutraceutical has no regulatory definition, being multi-targeted compounds with low-concentrations use [5,6]. Contrary to “functional foods”, considered food products fortified with vitamins, proteins, carbohydrates [7], the term nutraceutical refers to a substance, cultivated, produced, or extracted, that when administered to subjects is able to improve their health and well-being [8,9,10].

**Figure 1:**
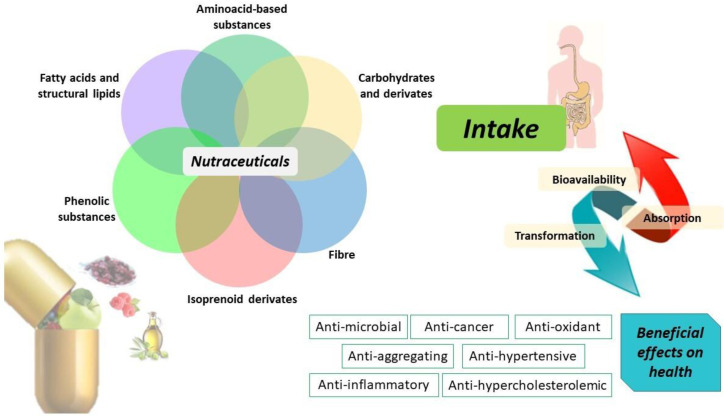
Nutraceuticals and their biotransformation improving human health.

Nutraceuticals are classified into different classes based principally on their chemical nature or mechanism of action, as shown in Figure 1. Therefore, nutraceuticals can include isoprenoid derivates, phenolic compounds, fatty acids, lipid, amino acids, fiber, and carbohydrate molecules capable of exerting specific therapeutic properties, such as antioxidant, anti-inflammatory, antimicrobial, and antineoplastic action [11].

The Mediterranean diet, Intangible Cultural Heritage of Humanity since 2010, is rich of nutraceuticals with health-promoting effects [12]. In fact, several studies have demonstrated that higher adherence to the Mediterranean diet significantly decreased the risk of metabolic chronic diseases, including cancer [12,13,14].

The exact mechanism by which the Mediterranean diet produces its beneficial effects is unknown. However, previous evidence has led us to distinguish several effects such as protection against inflammation, platelet aggregation, oxidative stress, and effective lipid lowering. Other effects consist of hormone modification and growth factors involved in cancer pathogenesis and the inhibition of nutrient detection pathways through the restriction of specific amino acids and gut microbiota-mediated metabolites that affect metabolic health [15,16].

These beneficial outcomes seem to also have important effects on the genetic patrimony of the single subject. For example, a higher adherence to the Mediterranean diet of older people gives different telomeric features, compared to those following a lower adherence [17]. The utilization of nutraceuticals is often associated with an increased interest and compliance to change lifestyle, so the Mediterranean diet becomes a nutritional style that allows the prevention and treatment of numerous metabolic diseases [18].

Recently, the field of nutraceuticals represents a very appreciated area of medicine, since more patients are interested or using dietary supplements. Interestingly, the impact of nutraceutical use on human health could have a synergistic effect with medications in improving metabolic parameters, i.e., blood pressure, cholesterol, and glucose. Nutraceutical options are numerous, and the advantages and disadvantages of each option according to the specific medical condition need to be objectively considered.

Clinical trials testing dietary supplements have observed beneficial changes mainly related to inflammatory mediators and adipokines [12]. In general, the polyphenol content is responsible for the health-promoting effects of foods, capable of exerting an anti-inflammatory, anti-hypertensive, anti-platelet, and antioxidant action [19,20]. Over the past few decades, polyphenols have become a field of interest for nutrition research due to their beneficial health effects. The polyphenols in green and black tea, grapes, and red wine have been intensively investigated, and scientific interest has recently grown in dark chocolate, another rich source of polyphenols such as theobromine, catechin, procyanidin B2, and epicatechin [21]. Researchers have reported that cocoa and chocolate–deemed a nutraceutical food–exerted positive effects on gastrointestinal motility [19,22,23]. Specifically, an in vivo study highlighted the prokinetic effects of dark chocolate on the gallbladder, resulting in gallbladder statis prevention.

On the other hand, one of the most important and difficult aspects of the study of nutraceuticals are to establish the best dosage without toxic consequences. This aspect is resolvable, considering their bioaccessibility, defined as the amount available for absorption in the gut, and their bioavailability, defined as the portion of the molecule absorbed and metabolized [24].

Moreover, phenolic compounds ingested with nutraceuticals undergo rapid metabolism in both enterocytes and the liver, whereas the non-absorbed phenolic compounds reach the colon, where they are subjected to extensive microbial metabolism [25]. The study of their effects must take into account the amounts of metabolites in plasma and tissues derived after absorption and digestion. Since not all metabolites can act as active compounds [26,27], the investigation of the quality of the metabolites produced is necessary to evaluate the efficacy of phenolic compounds, and consequently of the respective nutraceutical foods. Considering the high therapeutic value of dietary components on human health, this review attempts to summarize our research in the nutraceuticals area. Specifically, we included the main results obtained by in vitro and in vivo studies focused on extra virgin olive oil compounds, polyunsaturated fatty acids, and fruit components.

## 2. Role of Phytochemicals as Nutraceuticals

**Figure 2:**
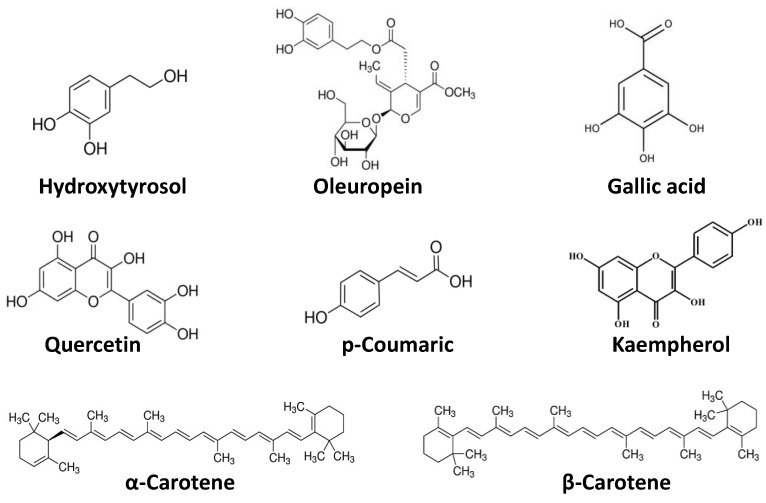
Chemical structures of common dietary phytochemicals.

Phytochemicals, considered chemical compounds naturally present in plants, give color, flavor, and structure. Although phytochemicals are not associated to nutritional functions, they play a key role as responsible compounds for multiple health benefits: to enhance the synthesis and activity of enzymes involved in the inactivation of carcinogens, suppress the growth of cancer cells, and interfere with the metabolic processes [28,29]. Therefore, phytochemicals could be defined as bioactive nutraceutical compounds [30,31]. Epidemiological evidence has shown that these substances act differently and synergistically, exerting overall protective effects against infections, tumors, diabetes, hypertension, and heart and cerebrovascular diseases. The abovementioned effects are due to the following actions: antioxidant, able to protect the body against oxidative stress; hormonal, for effects similar to those of natural estrogens; antimicrobial, through the enhancement of the immune system; hypolipidemic; interference with enzymatic activities and on DNA replication and modification, inactivating toxic substances, protecting DNA from the action of carcinogens and inhibiting the multiplication of cancer cells [32,33,34]. Phytochemicals can be classified into different classes (Figure 2). The most investigated consists of polyphenols–a heterogeneous group present in almost all plants–that comprise three classes of compounds: flavonoids or catechins (in onion, cabbage, broccoli, soy, tomatoes, fruit, wine, and tea with anticancer, anti-inflammatory, anti-hormonal, and antiplatelet effects); phenolic acids (in coffee and cocoa, with antioxidant and anticancer action), and phytoestrogens (in soy isoflavones and lignans of oleaginous seeds and whole grains, with antioxidant, anticancer and hypocholesterolemic action). Carotenoids–found especially both in orange and red fruits and vegetables–are a group of several compounds and natural pigments. They have been paid particular attention for their provitamin and antioxidant effects. The most well-known carotenoids are β-carotene, α-carotene, lycopene, and β-cryptoxanthin.

Thus, foods rich in phytochemicals include cereals, legumes, vegetables, fruits, and spices.

However, several studies have confirmed the absolute ineffectiveness and the potential danger of intake of these substances when indiscriminately and inappropriately used. In fact, high doses of these substances can interfere with other nutrients or be converted into substances with pro-oxidant action, thus becoming harmful substances [35,36].

## 3. Bioaccessibility and Bioavailability of Nutraceutical Compounds

Today, attention to beneficial food ingredients and nutraceuticals is growing, as confirmed by the new field of Foodomics [37]. Diet plays a very important role in the modulation of various metabolic functions. In fact, foods, in addition to providing the energy necessary for the normal metabolic processes of the body, are a unique source of “active ingredients” such as antioxidants, vitamins, polyunsaturated fatty acids, and fiber with beneficial effects on health. Thus, diet and its components can contribute to a state of well-being, to a reduction in the risks related to certain pathologies, and to an improvement in the quality of life [38]. There is growing attention to the nutritional value and risk of foods, as well as their possible beneficial effects on health, and there is an increasing need to provide correct information on the foods consumed every day. Foods consists of macronutrients (proteins, fats, sugars) that must be digested by releasing the respective monomers (amino acids, fatty acids, monosaccharides) and micronutrients (vitamins, mineral salts, polyphenols, dietary fiber). The presence of micronutrients in many cases increases the nutritional value of the food, as these compounds are often related to an increase in the body’s well-being [39]. Interest in understanding the relationship between the food–gut microbiota–health axis is steadily increasing because diet, lifestyle, and the environment affect the gut microbiota daily [40].

Specifically, Figure 1 depicts a classification of nutraceuticals into fatty acids and lipids, amino-acid-based substances, carbohydrates, fiber, isoprenoid derivates, and phenolic compounds that represent the most investigated classes of physiologically active compounds. These classes of nutraceuticals are mainly found in olive oil and in the fruit-based foods of the Mediterranean diet. The health benefits of ingesting phenolic compounds are strongly dependent on their bioaccessibility and bioavailability in the digestive tract and circulatory system. For this reason, an important aspect to consider is the bioavailability of these compounds which in turn depends on their bioaccessibility and intestinal absorption [41,42,43]. For example, gut microbiota contributes to the polyphenol biotransformation into metabolites and is modulated by the effect of polyphenols inhibiting pathogenic bacteria and stimulating beneficial bacteria with beneficial outcomes on host health. The intake of nutraceutical compounds potentially limits their absorption as the result of gastrointestinal fluids transporting them through the mucus layer, through epithelial cells [44]. However, when bioactive compounds are released from food and made soluble in gastrointestinal fluids, they interact with other system components exerting beneficial effects on human health (i.e., anti-inflammatory, anticancer, antioxidant, anti-hypercholesterolemic, anti-hypertensive properties).

Examples of bioavailable nutraceutical compounds are microencapsulated bioactive molecules, such as flavonoids, phenolic compounds, antioxidant molecules, carotenoids, and plant metabolites in general. In fact, micro/nano-encapsulation processes are applied to protect, stabilize, increase bioavailability, and control the release of active ingredients such as pigments, antioxidants, vitamins, minerals, peptides, and proteins. Therefore, the encapsulation of nutraceuticals allows them to protect and interact with the gastrointestinal tract and to increase their solubility by improving their bioavailability [45,46,47].

## 4. Extra Virgin Olive Oil Compounds

**Figure 3:**
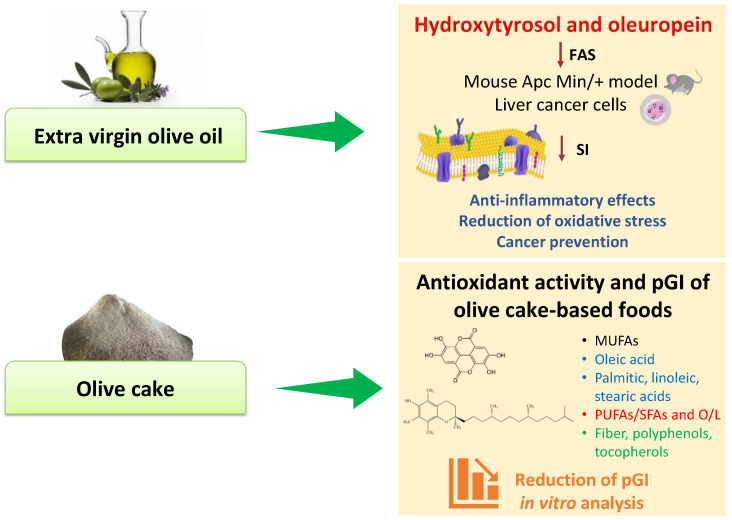
In vitro effects of extra virgin olive oil and olive cake. Abbreviations: FAS, fatty acid synthase; L, linoleic acid; MUFAs, monounsaturated fatty acids; O, oleic acid; pGI, predicted glycemic index; PUFAs, polyunsaturated fatty acids; SFAs, saturated fatty acids; SI, saturation index.

Extra virgin olive oil is the product extracted from the fruit of the olive tree (*Olea europaea* L.) rich in bioactive compounds [48], recognized to exert healthy properties with different effects on cell biology. Although extra virgin olive oil has a high concentration of oleic acid, its minor components, principally the phenolic compounds, appear to be responsible for the greatest number of its beneficial effects. Moreover, the profile and concentration of these olive oil minor components depend on the cultivar [49], irrigation [50], and type of cultivation [51], all factors which can modify the expression and the content of polyphenols such as oleuropein and hydroxytyrosol. Hydroxytyrosol comes from the hydrolysis of oleuropein that is mostly present in the fruit and in the olive leaves [52]. It has shown strong antioxidant effects with capability to scavenge oxygen and nitrogen free radicals. Different experimental studies have demonstrated that hydroxytyrosol has anticancer properties exerting pro-apoptotic effects [53,54,55,56,57]. Moreover, this promising molecule can modulate the expression of several genes involved in cell proliferation and apoptosis [58]. The cell antiproliferative effects of hydroxytyrosol and oleuropein are sustained by the inhibition of fatty acid synthase activity [59] that is crucial for the action of extra virgin olive oil in the cells. Fatty acid synthase activity levels, the key enzyme in the fatty acid’s biosynthesis pathway and its mRNA expression, are downregulated after olive oil treatment in an ApcMin/+ mouse model [60], as depicted in Figure 3.

Moreover, in the same animal model of colon carcinogenesis, we demonstrated that olive oil treatment was able to counteract the intestinal polyp growth and to control the environmental conditions in which tumors develop [61,62]. The intestinal cell proliferation was reduced through the inhibition of fatty acid synthase and hydroxy-3-methylglutaryl-CoA reductase (HMGCoA reductase), which is the rate-limiting enzyme for cholesterol synthesis [61]. In further studies, we evaluated other mechanisms by which olive oil could reduce cell proliferation and increase apoptosis in mice intestinal polyps: (1) the decrease of Phospho-Stat3-Ser727 (p-STAT3 Ser), known to be responsible for the activation of metabolic pathways involved in regulation of cell proliferation, and (2) the increase of the expression of estrogen receptor β (ERβ) and consequently of ERβ/ERα ratio, already known to be a diagnostic and prognostic parameter for colon cancer progression [62,63,64,65].

A lipidomic approach has also allowed us to investigate the effect of olive oil intake on tissue fatty acids profile in ApcMin/+ mice [66]. Extra virgin olive oil affects the saturation index of the cell membrane, defined as the ratio of stearic acid to oleic acid [67]. The saturation index, considered an indicator of cell membrane fluidity, is a crucial parameter to assess the healthy status of the cell because of evidence that low levels of SI are associated with the cell malignant phenotype [68,69]. In addition, alterations of the saturation index are often associated with changes in the hydration levels of cell membranes and with the activation of proteins involved in cell growth and proliferation.

The extra virgin olive oil anti-inflammatory effects on the gut mucosa have also been demonstrated in humans [70,71]. The daily consumption of extra virgin olive oil has been considered useful for the prevention and management of intestinal diseases, such as inflammatory bowel diseases (IBD) [72].

In assessing the well-known and marked properties of virgin olive oil components, a new scenario consists in a valorization of a by-product from extra virgin olive oil extraction, namely olive cake, a favorable source of bioactive molecules. In a previous study, we evaluated the chemical and nutritional characterization of olive cake in terms of saturated fatty acids (SFA), monounsaturated fatty acids (MUFAs), polyunsaturated fatty acids (PUFAs), total dietary fiber, PUFAs/SFA, oleic/linoleic acid (O/L), phenolic content, and antioxidant activity [73]. Although olive cake represents a waste product of the olive oil production, it still displayed strong antioxidant activity—as measured by ABTS and DPPH assays—and high total phenol content, especially polyphenols and tocopherols. For these reasons, a new perspective was to use olive cake as a favorable raw material for the enrichment of gluten-free breadsticks’ nutritional value.

Notably, scientific nutritional research has clearly demonstrated that diet and its components influence the state of health, modulating favorably or unfavorably aspects of the physiology (i.e., the functioning) of our organism [74,75]. Therefore, it is important to know not only the composition of foods, but also the effects that the different components produce in the body. It has been suggested that low-glycemic-load diets improve weight and fat mass loss compared to high-glycemic-load diets, and that reducing overall glycemic load is beneficial as an adjuvant in appetite regulation, in maintaining weight loss, and in controlling weight [76,77,78]. Even some important parameters of cardiovascular risk (such as triglycerides and HDL cholesterol) and the parameters of the “inflammatory state” of the organism, linked in multiple ways to the state of health, are favorably affected by the consumption of low-index foods and glycemic load.

In contrast, a diet rich in foods with a high-glycemic index is associated with the increase in circulating triglyceride levels and the reduction of HDL-cholesterol with a greater risk of suffering from type diabetes 2 due to the excessive secretion of insulin and the consequent functional loss of pancreatic cells induced by their consumption [79]. In this scenario, the in vitro starch hydrolysis of products fortified with bioactive molecules of olive cake showed a reduced predicted glycemic index (pGI) as a result of fermentation processes by lactic acid bacteria (Figure 3) [73]. Other studies encompassed the mechanisms of olive cake polyphenols in protecting the hepatocytes and endothelial cells against triglyceride accumulation and oxidative stress [80,81]. Moreover, a strong correlation was observed between olive oil polyphenols consumption and gut microbiota. Microorganisms populating the gut microbiota are involved in the transformation and bioaccessibility of olive oil polyphenols into molecules that modulate the imbalance of the gut microbiome, reducing liver oxidative stress and inflammation [82,83].

## 5. Polyunsaturated Fatty Acids

Diet provides a high value of fats representing a substantial portion in most of all foods. However, it is important to distinguish PUFAs from SFAs because many of the positive effects of fats on human health have been attributed to PUFAs [84,85]. PUFAs are those involved in the proper functioning of the organism’s biological activities, and it is currently recommended to increase PUFAs compared to SFAs [86,87]. Several foods are rich in PUFAs, both vegetable and animal, such as olives and olive oil, fish, soy, corn, sunflower, nuts, and sesamum and other seeds [73,88,89,90,91].

The beneficial effects of long-chain PUFAs of the n-3 and n-6 (n-3 PUFAs and n-6 PUFAs) in human health are well-known, as well as the importance of having a lower n-6/n-3 PUFAs ratio in the diet [92,93]. However, the current dietary patterns, mainly in Western countries, result in an excessive intake of n-6 PUFAs with a consequent n-6/n-3 PUFAs unbalanced ratio [94]. Actually, the recommendations suggest that this ratio should not exceed 4:1, preferring foods with a lower content of n-6 PUFAs.

Regarding PUFAs, the major precursors, linoleic acid (C18:2n-6, LA) and α-linolenic acid (C18:3n-3, ALA), cannot be synthesized by humans and consequently need to be acquired directly from the diet [94].

The most physiologically representative n-3 PUFAs family are eicosapentaenoic acid (C20:5n-3, EPA) and docosahexaenoic acid (C22:6n-3, DHA), whereas the n-6 PUFAs series is mostly characterized by arachidonic acid (C20:4n-6, AA).

Several epidemiologic and intervention studies highlighted that n-3 PUFAs have anticancer properties, inhibiting colon carcinogenesis and affecting tumor progression [95,96,97]. In previous in vitro studies, it has been demonstrated that n-3 PUFAs arrest cell growth and promote cell apoptosis [98,99]. EPA and AA exerted a growth inhibition of HepG2 cells associated with the downregulation of gene expression of lipogenic enzymes such as 3-Hydroxy-3-Methyl-Glutaryl Coenzyme A Reductase (HMGCoAR) and fatty acid synthase. In liver cancer, PUFAs may control the gene expression of these enzymes through SREBP-dependent regulation pathways [99]. In addition, the beneficial effects of PUFAs are also recognized due to their role in activating peroxisome proliferator activated receptors (PPARs), as shown in other human tumor cell lines [100].

Pre-clinical studies have shown that a diet rich in n-3 PUFAs is able to control the intestinal polyp formation in mice [60,66,101]. A diet rich in n-3 PUFAs is known to contribute to modulating intestinal polyp formation. However, the ApcMin/+ mice fed n-3 PUFAs also showed a reverse mechanism of polyps development through the overexpression of the estrogen receptor β and low density lipoprotein (LDL) receptor, known to be negative modulators of cell proliferation. Another mechanism sustaining the antiproliferative action of n-3 PUFAs is the modulation of CB1 receptor expression [102]. In the ApcMin/+ mouse model, dietary intake of n-3 PUFAs significantly inhibited intestinal polyp growth, also inducing CB1 receptor gene and protein expression. The upregulation of this receptor was associated with the inactivation of the Wnt/β-catenin pathway, affecting cell growth and proliferation [102].

## 6. Fruit Components

Nutrition experts continually emphasize the importance of fruit in the diet. The Mediterranean diet places fruit and vegetables as the basis of a daily diet regime because of their significant content of vitamins, mineral salts, and bioactive compounds beneficial for human health [20,103]. Table grapes, in particular, are a typical fruit of the Mediterranean tradition and consumed all over the world. Grape (*Vitis vinifera* L.) is a fruit mainly rich in polyphenols, molecules able to prevent cancer, reduce tumorigenesis, and influence cell proliferation-related pathways.

**Figure 4:**
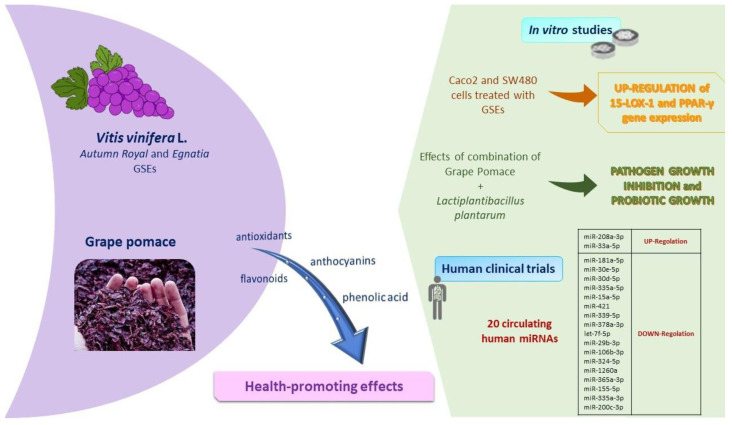
Main features of fruit components: summary of in vitro and in vivo studies performed on *Vitis vinifera* L. and grape pomace. Abbreviations: 15-LOX-1, 15-lipoxygenase-1; GSEs, grape skin extracts; miRNAs, microRNAs; PPAR-γ, peroxisome proliferator-activated receptor-γ.

The polyphenolic composition of grapes, which depends on genotype, environmental factors and agronomic practices [104], affects the structural components of the cytoskeleton, cellular adhesions, and the cell membrane fatty acids profile [104]. Briefly, Figure 4 shows the main results obtained by our studies on grape skin and grape pomace extracts, as below. Recently, we focused on the biochemical properties of two table grape skin extracts, Autumn Royal, a seedless black grape and Egnatia, a new red seedless genotype obtained by breeding programs carried out by the Research Centre for Viticulture and Enology of the Council for Agricultural Research and Economics based in Turi, Italy. Particularly, the two grape skin extracts modulate cell proliferation differently in human colorectal cancer cell lines [105]. Table grape Egnatia has shown an increased ability to influence cell proliferation and apoptosis, as well as to exert a growth arrest in the S phase of the cell cycle, particularly in the Caco2 cell line [105].

Because Autumn Royal was characterized by higher antioxidant activity compared to Egnatia, Autumn Royal grape skin extracts, significantly at low doses, blocked cell migration and exerted morphological changes of the cultured cells [106]. These extracts regulated cell migration through the stimulation and formation of polarized lamellipods, modulating cytoskeletal organization and inducing characteristic changes, such as the reduction of cell cytoplasm and the decrease of surface microvilli [106].

Moreover, the lipidomic analysis of cell membranes showed that two grape skin extract polyphenols affected membrane fluidity by influencing its content of PUFAs. These data demonstrated that the antitumor mechanism of two grape skin extracts in vitro involves membrane PUFAs composition and their downstream pathways.

During the tumorigenesis of the colon, the oxidative metabolism of PUFAs is regulated by different enzymes, and the 15-lipoxygenase-1 (15-LOX-1) play a key role in exerting antioxidant and antimetastatic action through the activation of peroxisome proliferator-activated receptor-γ (PPAR-γ). The results of in vitro studies highlighted that Autumn Royal and Egnatia grape skin extracts influenced the gene expression of 15-LOX-1 and PPAR-γ. In particular, grape skin extracts polyphenols induced a significant upregulation of both 15-LOX-1 and PPAR-γ [104]. Together, these activated factors are required for the inhibition of the colorectal tumorigenesis process.

Although the biological effects of grape skin extracts could depend on the type of cancer cell, certain flavonoids and non-flavonoids compounds contained in grapes can act synergistically to provide particular antiproliferative effects on cancer cells.

Not only grapes, but also grape pomace exerted important outcomes evaluated in the in vitro experiments conducted in our previous studies. Grape pomace is a major by-product obtained from the winemaking process that contain phenolic compounds with beneficial effects on human health. Recently, we evaluated the antioxidant effects of grape pomace digested extracts [107], evaluating in vitro models their prebiotics and probiotic activities. Previous results showed the capacity of bacteria to grow in the presence of polyphenols as they metabolize them. A positive correlation between bacteria effects and the modification of pH during gastrointestinal digestion was observed; in fact, in the presence of digested grape pomace extracts, Gram-positive (Bacillus megaterium and Listeria monocytogenes) and Gram-negative (Escherichia coli) bacteria were inhibited, while a probiotic Lactobacillus plantarum grew, as shown in Caponio et al. [107]. In line with these results, our group is performing a study on the anticancerogenic effects of digested grape pomace extracts in human colon cancer cell lines. Preliminarily unpublished results showed that polyphenols in digested grape pomace extracts inhibited cell proliferation already starting from 25 µg/mL, both after 24, 48, and 72 h of treatment with dose-dependent effects. These results shed light on an interesting view for the conceptualization of functional food ingredients or the formulation of food supplements based on these extracts.

An interesting alternative to grapes is represented by oranges that have known beneficial properties for health [108]. This fruit is rich, mainly in naringin and hesperidin, polyphenol compounds known to interfere with cholesterol intestinal absorption [109]. The regular consumption of orange exerts an anti-hypercholesterolemic action through an upregulation of the LDL receptor [110]. In vitro studies reported the inhibitory activity of two glycosides, naringin and hesperidin, against hydroxymethylglutaryl-CoA reductase (HMGCoAR), the key enzyme in the cholesterol biosynthesis in the liver [111].

Furthermore, the whole extract of orange has been demonstrated to affect blood lipid profiles in subjects with metabolic syndrome [112], as well as to modulate serum levels of lipase and amylase enzymes [113].

Recently, new experiments are being conducted with liver cancer cell lines to evaluate the combined effect of naringin and hesperidin on cell growth and proliferation. In these treated cells, possible changes in the lipid and glucose metabolism will be studied. Unpublished data have demonstrated that orange polyphenols are able to affect cell membrane fatty acids profile, suggesting an interesting nutraceutical potential of orange in subjects with metabolic diseases. Moreover, scientific evidence reported the promising bioactive effects of *Spondias mombin* L.—fruiting species of the Anacardiaceae family—in the regulation of gastrointestinal inflammation due to its contribution in terms of antioxidant activity and gastric mucus production [114]. In addition, in vivo studies on rats with oral mucositis highlighted the anti-inflammatory effects of the same extract. In fact, *Spondias mombin* L., due to the phenolic compounds (especially ellagic and chlorogenic acid), inhibited leukocyte migration [115].

Another molecule with high potential in the regulation of inflammation is the extract of Caryocar coriaceum Wittm fruits, as confirmed in a recent study [116].

## 7. Future Perspectives and Conclusions

To date, the demand for health care has intensively increased, and the attention of people has focused on the requirement for foods enriched with nutraceuticals to obtain adequate nutrients that allow both to maintain the normal functioning of the body and to prevent disease. Based on experimental studies, the therapeutic purpose of nutraceuticals depends on their bioavailability, solubility, toxicity, and delivery system [117].

Recent studies have demonstrated that dextrins can be used in the nutraceuticals delivery industry because of their non-toxic, biodegradable, and water solubility characteristics [117,118]. Dextrins—derived from starch—are considered an effective drug delivery tool with proven clinical tolerability [119], and the dextrin polymer-based nanocarriers are designed to be the preferred and natural system for providing nutraceuticals.

The main purpose of delivery studies is to make the ingested nutraceuticals accessible for the intestinal tract. This item is important as the main effects of these compounds occur in gastrointestinal system.

Further studies will be launched using the in vitro and in vivo experiments on the interaction of nutraceutical-loaded nanoparticles with human organs, tissues, and cells, as well as the evaluation of their effects on specific tissue metabolism. The use of nanotechnology in health care is emerging with excellent results, and the research in biomaterial design for nutraceuticals delivery is becoming widespread.

These studies will also be important to deepen the knowledge of the bioaccessibility of nutraceuticals foods, since this information allows for the design of the most appropriate combinations of bioactive molecules in the treatment or prevention of diseases.

In conclusion, the knowledge of the specific effects of diet components can be useful in the prevention and treatment of several diseases, including gastrointestinal disease and cancer. In the scenario, this review provides more in-depth knowledge on the effects linked to nutraceutical administration on human health, and therefore, it is aimed to suggest the use of a specific dietary component for personalized adjuvant therapies.

## Data Availability

Data is contained within the article.
